# A Blockchain and Fingerprinting Traceability Method for Digital Product Lifecycle Management

**DOI:** 10.3390/s22218400

**Published:** 2022-11-01

**Authors:** Jose Luis Gonzalez-Compean, Victor Jesus Sosa-Sosa, Jose Juan Garcia-Hernandez, Hiram Galeana-Zapien, Hugo German Reyes-Anastacio

**Affiliations:** Centro de Investigación y de Estudios Avanzados del I.P.N. (Cinvestav Tamaulipas), Victoria City 87130, Mexico

**Keywords:** DPLM, blockchain, fingerprinting, digital value chain, security

## Abstract

The rise of digitalization, sensory devices, cloud computing and internet of things (IoT) technologies enables the design of novel digital product lifecycle management (DPLM) applications for use cases such as manufacturing and delivery of digital products. The verification of the accomplishment/violations of agreements defined in digital contracts is a key task in digital business transactions. However, this verification represents a challenge when validating both the integrity of digital product content and the transactions performed during multiple stages of the DPLM. This paper presents a traceability method for DPLM based on the integration of online and offline verification mechanisms based on blockchain and fingerprinting, respectively. A blockchain lifecycle registration model is used for organizations to register the exchange of digital products in the cloud with partners and/or consumers throughout the DPLM stages as well as to verify the accomplishment of agreements at each DPLM stage. The fingerprinting scheme is used for offline verification of digital product integrity and to register the DPLM logs within digital products, which is useful in either dispute or violation of agreements scenarios. We built a DPLM service prototype based on this method, which was implemented as a cloud computing service. A case study based on the DPLM of audios was conducted to evaluate this prototype. The experimental evaluation revealed the ability of this method to be applied to DPLM in real scenarios in an efficient manner.

## 1. Introduction

The global digital content market will grow about 30% from 2020 to 2025, with a trend to the distribution of digital content through content delivery networks (CDNs) according to [1]. Technology trend analyses reveal that blockchain and cloud computing are two cornerstone disruptive technologies for managing digital products during the stages of their lifecycle [2]. This digital product lifecycle management (DPLM) includes stages such as manufacturing, retailing and delivery of digital products [3]. The verification of the accomplishment/violations of agreements defined in digital contracts is a key task during the DPLM [4]. This verification becomes more relevant when validating both the integrity of digital product content and the transactions performed during multiple stages of the DPLM (e.g., manufacturing, retailing and delivery) among different organizations [4].

Blockchain is a popular traceability tool defined by Nakamoto in [5] based on data mining and bitcoin techniques to develop data structure and encode the transaction of information. As described in [6], blockchain technology (BCT) is a distributed digital ledger that allows one permanently to record transactions of all type of assets (multimedia data, finance, software, ideas, etc.), with high transparency and security, making it impossible to change, hack or cheat. BCT has shown rapid growth in supporting supply chain traceability, sustainability and information security over the last decade [7].

The popularization of online distribution platforms for digital content exposes the need for robust technologies to guarantee the source and and originality of these products [8]. However, the risk of the digital product being copied for unauthorized use or being required for an offline verification in the case of a dispute or scenario involving an agreement violation exists during the various phases of a supply chain or throughout the DPLM.

To address this concern, promising techniques have emerged such as traitor tracing, also known as fingerprinting, transactional watermarking, content serialization or user forensics [9]. These techniques aim to identify the origin of a leak in a distribution framework, acting as an incidence response mechanism to point at the violation source. The BCT is basically applied online, whereas fingerprinting is commonly used in an offline manner. Both techniques and strategies can complement each other for organizations to establish the integrity validation of a digital product in DPLM and the validation and traceability of the transactions performed with it, which, however is not trivial as both technologies are not integrated and are both consuming computing tools.

This paper describes a traceability method for DPLM based on the integration of online verification (blockchain) and offline verification (fingerprinting) by using conceptual abstractions that represent the key elements of this method. On the one hand, BCT offers characteristics that benefit digital product traceability as was proven in [10]. On the other hand, collusion-resistant fingerprinting is a practical solution to the tampering detection in digital content as it allows media owners to detect any unauthorized digital content and trace it back to the dishonest users, as studied in [11].

In our proposed method, a blockchain lifecycle registration model is used for organizations to register the exchange of digital products with partners and/or consumers in the cloud through the stages of a DPLM as well as to verify the accomplishment of agreements at each stage. The Sha256 algorithm is used as a method in the smart contracts to create the hashes of the input and outcomes as well as to produce the hash of all the information stored in the block chains (input and outcome hashes, timestamps and information required by smart contracts). The integrity is verified in two phases: in the first one, the hashes of the inputs and outcomes are registered both in the blockchain and within the contents by using the fingerprinting method. The verfiability process is thus supported by an integrity service at the registration phase and by the blockchain querying at the online phase as well as by the fingerprinting at the offline phase. In this way, the organizations associated with a given stage can verify the accomplishment by recovering the contents from the origin (by using input paths) and outcomes for comparing the hashes (input/outcome hashes) with those registered in the blockchain or those registered within the contents (by using fingerprinting). This result is useful in either dispute or agreement violation scenarios and when users have no access to the whole trace of the DPLM. In a similar way, information encrypted in digital envelopes is used to verify value properties registered both in blockchain and the contents (e.g., timestamps, content sizes, etc.) and to compare this reference information with that registered in the smart contracts (e.g., min delivery time, max delay allowed, etc.).

Furthermore, an implementation of a DPLM prototype as a cloud serverless service, based on the given verification approach, is also described in the paper. To evaluate this prototype, a case study based on the DPLM of digital audios was carried out. An experimental evaluation of the implementation of this prototype was conducted to validate the feasibility of the cloud service. Encouraging results show the ability for this method to be applied to DPLM in real scenarios in an efficient manner.

## 2. Related Work

In this section we describe the main research efforts found in the literature targeting the development of traceability approaches in supply chains. We concentrate on BCT in supply chains, tampering detection, as well as identification and location tracking technologies.

### 2.1. Blockchain-Based Supply Chains

Nowadays, we can find different research and development projects that demonstrate the benefits of BCT when implemented in the supply chain management [10,12,13]. In general, BCT is used for creating a permanent, shareable, actionable record of every moment in the journey of digital products through the supply chain. How to ensure product validation and achieve efficient traceability are requirements that could be addressed by BCT in current supply chains [14]. Despite its importance, just a few papers deal with traceability in non-finance applications that integrate BCT [10,15,16].

The difference between the conventional supply chain and integrated blockchain-based supply chain was underlined in [17]. Authors highlight the problems faced by the automotive industries, pharmaceutical industries, food industries and retail industries by using the conventional supply chain as well as the solutions to the problems using blockchain-based systems. It was observed that the integrated blockchain-based supply chain overcomes the problems and makes the system more efficient and trustworthy. A traceability system of steel products by using blockchain-based industrial Internet of Things (IoT) was proposed in [13]. In the system, production companies, logistics and consumers can participate in the information certification of steel products. Another product traceability system based on BCT was presented in [18], in which all product transferring histories are perpetually recorded in a distributed ledger by using smart contracts and a chain is formed that can trace back to the source of the products. A blockchain-based framework using Hyperledger Fabric [19] to ensure the traceability of electronic parts in the supply chain using a unique device identification (ID) is introduced in [20]; the authors conclude that additional research is needed to explore the use of the unique ID obtained for a physically unclonable function (PUF), which could help to link the physical device to the blockchain in a tamper-resistant manner. The work in [21] proposed a system for asset tracking using blockchain whose objective is to trace the route of the products in the supply chain, making sure it is not tampered with and if it is, then indicating exactly where the tampering took place in the supply chain.

According to the high-level reference architecture in [22], security services must encompass three different levels of the blockchain, namely, process, data and infrastructure. Following, we describe general security concerns of BCT. The smart contracts at the process level could be vulnerable to security threads such as reentrancy and unpredictable states (code vulnerabilities). At the data level, the privacy and fine-grained access control can be met by means of smart contracts and attribute-based encryption (ABE) schemes. Many different access control schemes have been proposed in the literature for BCT; a difficult research question tackled is how to effectively withdraw access that has already been granted [22]. Other research issues such as reduced overheads, latency and increased availability are of particular interest in integrated BCT and IoT systems [23]. Additionally, the BCT leverages homomorphic encryption to improve the security of the system against attacks on user wallets, preimage attacks and collision attacks. Lastly, infrastructure-level security faces challenges of private key management, terminal and networking vulnerability and compliance enablement.

### 2.2. Tampering-Resistant Approaches

Independent projects, without an underlying BCT, have addressed the problem of tampering with digital content by means of using fingerprinting [24,25], as previously introduced. This technique consists of hiding information in each copy of the distributed content, in which potential unauthorized copies of a digital product are equal, but the hidden information make them different from one user to another. Collusion attack is a typical attack in fingerprinting systems, where a group of users combine their copies in order to remove the original fingerprint. If a sufficient number of copies are combined, the noise produced by the collusion attack can disable/confuse the fingerprint detector and prevent the content owner from identifying the illegal users. Different solutions to this problem have been proposed using collusion-resistant fingerprint codes [11,25,26,27,28]. Theoretical results for collusion-resistant fingerprint codes have shown interesting properties against collusion attacks; however, in practical scenarios their performance needs further research as these can be sensible to other kinds of attacks [29].

Most literature mainly considers the business process modelling and the technology design process of a blockchain-based solution and the reference frameworks’ lack in terms of standard methodology concerning designing, developing and validating an overall solution for verification of DPLM [16]. In recent years several prototypes have been developed using BCT; some of them are implementations of IBM [30]. Application-specific implementations, such as Filament for IoTs, or Mediachain (acquired by Spotify in 2017) for smart contracts have emerged. However, concrete end-to-end implementations with detection of users tampering with digital products are unavailable as of this writing, providing very limited evidence for us to assess the impact of BCT on supply chains in a scenario solution for verification of DPLM. In this context, our paper presents an implementation of a blockchain and fingerprinting method in a DPLM prototype in an attempt to fill this void.

### 2.3. Traceability Enabling Technologies

There are some techniques and technologies used to store information about products and to track their physical location and current status throughout the supply chain stages. In particular, barcodes, QR codes and radiofrequency identification (RFID) have been successfully used to support traceability methods in different use cases [31]. In this regard, RFID-based traceability solutions are commonly preferred for indoor location identification due to a number of advantages: (a) RFID supports no-line-of-sight between the tag and the reader; (b) greater read range than barcodes; (c) RFID tags have large memory to store product’s information; and (d) it supports multiple readings simultaneously. However, RFID passive tags show slow efficiency when reading the tags in the presence of certain materials (e.g., liquid material or metals) that negatively influenced the radio wave propagation back to the reader [32]; in such cases, barcoding approaches could be more suitable. Furthermore, other technologies like the global positioning system (GPS), wireless sensor networks (WSN) and IoT can support traceability tasks at different stages of supply chains. Specifically, GPS technology provides accurate outdoor location tracking and it can be utilized for asset traceability over large service coverage areas. On the other hand, WSN and IoT sensors are useful to continuously sense, collect and transmit physical properties of the product of interest, such as temperature and humidity in drug management [33] and food supply chains [34,35].

## 3. A Blockchain and Fingerprinting Traceability Method for DPLM

**Figure 1:**
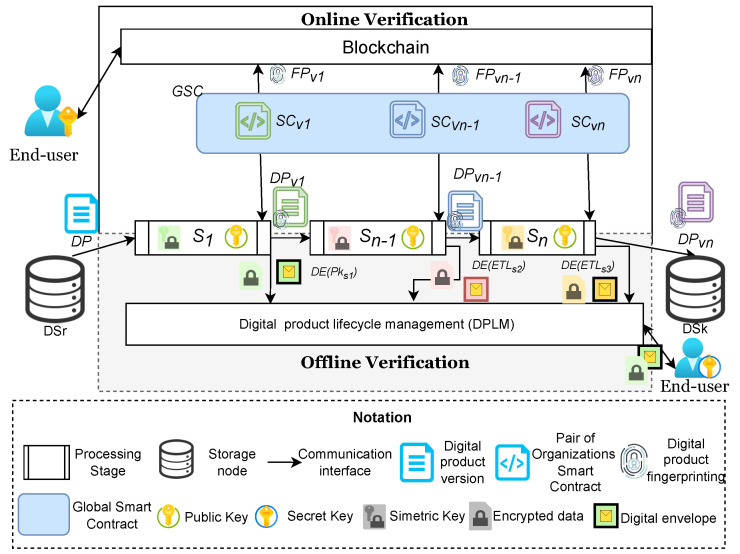
Digital product lifecycle management with online and offline verification.

**Figure 2:**
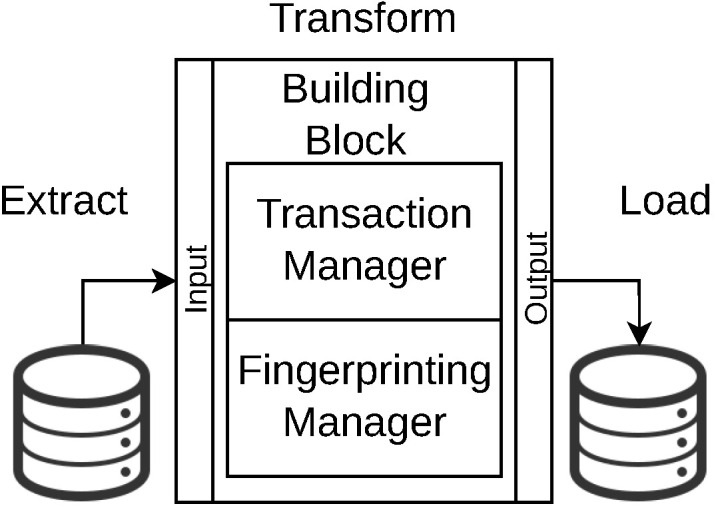
ETL metadata capturing in a building block.

We propose a blockchain and fingerprinting traceability method for DPLM, which is depicted in Figure 1. This method considers encapsulating each application in a pipeline used in DPLM into building blocks ($S_{1},\ldots ,S_{n-1},S_{n}$), which are managed as directed acyclic graphs to create digital value chains (see an example of *n* stages in a DPLM in Figure 1). The pipeline is expressed as:

$$Pipeline=(DSr,Stages_{i}^{n},ETL_{patt},DSk)$$

where DSr is a data source from which the original content is read, $Stages=\{S_{1},\ldots ,S_{n}\}$ is a set of *n* processing stages, all the stages are described with the extract, transform and load (ETL) model illustrated in Figure 2. The load process of a stage, $S_{i}\left[out\right]$, is connected to the extract process of the next stage, $S_{i+1}\left[in\right]$ and each stage has a transformation task that adds required characteristics to the data, $S_{i}\left[task\right]$. The DSk represent the final data sink where the processed data are preserved.

Each building block includes subblocks for managing applications, data inputs and outcomes (see Figure 2). The application management subblock manages the applications used at each stage of a DPLM. This subblock also includes two managers: the first one for registering transactions performed by that block. This manager includes two types of registration; the first one is a mechanism connecting each building block with the blockchain to perform transaction registration (see, in Figure 1, the registration of transactions performed between $S_{1},\ldots ,S_{n-1}$ in the form of a register as $FP_{V1}$), which should be exact with the characteristics described in the smart contracts registered in the blockchain and defined at the DPL Manager (see the DPL Manager in Figure 1). The second registration mechanism is based on a fingerprinting mechanism, which inserts the very register sent to the blockchain within the digital product (see a finger printing of digital product $DP_{V1}$ in Figure 1). The input including extraction tools and outcome (loading data to the next block or sink) subblocks are in charge of managing the data reaching and departing each stage of a DPLM (see extract and load connectors within building block in Figure 2).

The DPLM component includes two schemes for the verification of agreements or violations of smart contract specifications. The first one is an online mechanism that basically monitors blockchain registers and compares the events with the actions related to the smart contracts created for a DPLM. The second one is an offline mechanism that end users can use to verify any version of digital products created during the DPLM. This mechanism basically receives a digital product version and recovers the fingerprinting message, which describes the transactions performed over that digital product at its last stage of its DPLM. For instance, the fingerprinting message recovered by processing $DP_{V1}$ will describe the transactions performed by $S_{1}$ and $S_{n-1}$ for that asset ($DP_{V1}$ in Figure 1).

The online verification, based on the blockchain querying mechanism, enables end-users to keep monitoring the smart contracts of whole DPLM, whereas the offline version enables end-users to identify violations at DPLM stage level. These mechanisms will be described in the next sections.

### 3.1. Definition of Smart Contracts for DPLM Pipelines

In this traceability method, the applications used during a DPLM are modeled as stages in a value chain. A stage is an application, service, function or method that transforms a digital product into a new value-added version. A value chain is a sequential and orderly set of stages, each of which adds value to the digital product. The dataflow throughout a value chain from raw material to final product represents a DPLM pipeline.

The processing of stages is modeled by using the ETL model, as shown in Figure 2. This model produces a metadata scheme for capturing the context in which a digital product has been processed/managed by each stage. The extraction is the information about the digital product delivered in a previous stage (or obtained from a data source). The incoming digital product is processed (transformed to add value to that digital product) and *loaded* to another stage or another data source or sink. This ETL metadata are used to create a smart contract where the stages represent the organizations in that contract, whereas each digital product exchanged between organizations is an *asset* considered in a *transaction* by organizations (stages). The ETL information represents the information describing the transactions performed over the assets exchanged by organizations (considered in a smart contract) and it is defined as follows:

$$DPL_{DP_{V_{i}}}=Hash_{DP_{V_{o}}}∥Hash_{ETL_{Stage_{i}}}\left(DP_{V_{i}}\right)$$

where $DPL_{DP_{V_{i}}}$ represents a transaction producing the *i*-th version of a digital product lifecycle (DPL), which has been produced from an original digital product ($DP_{V_{o}}$). This is captured by a smart contract as the $ETL_{Stage_{i}}$ has registered the *extraction* (the data source path), the *transformation* performed by $Stage_{i}$ ($Hash_{DP_{V_{i}}}$) and the *loading* describing the path of $Stage_{i+1}\vee dataSink$ (e.g., either data lake or data warehousing).

The description of a DPL thus is basically the chaining of ETL metadata for all the stages managing a given digital product ($DPL_{DP_{V_{i}}}$) or even the original product ($DPL_{DP_{V_{o}}}$), which is represented by the following scheme:

$$DPL_{DP_{V_{o}}}=DPL_{DP_{V_{i}}}∥DPL_{DP_{V_{i}-n}}∥DPL_{DP_{V_{n}}}$$

### 3.2. Online Verification of Transactions by Using Blockchain

Blockchain is a sequence of blocks that contains a complete list of transaction records analogous to a conventional accounting ledger. This chain is distributed, transparent, immutable and uses consensus protocols. A blockchain block is built by metadata describing the transactions such as identifiers of blocks (i.e., a unique code in the form of a hash) and date and, in our method, each transaction performed in each stage of a DPL ($DPL_{DP_{V_{i}}}$) is registered in the blocks of this blockchain network in the form of a chain of ETL hashes and the information about the participants in these transactions is also registered into the blocks of the blockchain network by using a registry method (see Algorithm 1). As a result, each block produces enough information to verify the accomplishment/violations of sets of a pair of organizations’ smart contracts ($SC_{i}$) in cases of the DPL being performed by a set of organizations. If all the stages are executed for the same organizations then a global smart contract (GSC) is produced (see GSC in Figure 1) instead of a set of $SC_{i}$. In order to provide confidentiality, an AES-based CBC process is used to encrypt the metadata of the ETL, each stage produces a symmetric key for each file. To provide access control, this key is shared by using a digital envelope. This technique requires that each user (organization) creates public and secret keys; the public key is shared with another user ($S_{i}$). When $S_{i}$ requires the sharing of sensible data (e.g., a symmetric key), it encrypts this data with the public key of the organization and only the organization with the complementary private key can extract the content. For this prototype, a digital envelope is generated for each transaction and organization in the DPLM. This could produce performance and scalability issues if a large number of organizations were included in the DPLM. We are working on the incorporation of an attribute-based access control technique to prevent these potential issues.
**Algorithm 1** Online verificability process**Require:**N≥0,DSrPath,DSkPath,OrgPubKey,OrgId$n=0,DP_{V_{n-1}},DP_{V_{n}},DP_{V_{n+1}}$**while**n<N**do**    **if** n==0 **then**        $Stg_{n}\left[E\right]=ExtractFromDSr\left(DSrPath\right)$        $Stg_{n}\left[L\right]=LoadTo\left(Stg_{n+1}\left[E\right]\right)$    **else if** n==(N−1) **then**        $Stg_{n}\left[E\right]=ExtractFromStg\left(Stg_{n-1}\left[L\right]\right)$        $Stg_{n}\left[L\right]=LoadTo\left(DSkPath\right)$    **else**        $Stg_{n}\left[E\right]=ExtractFromStg\left(Stg_{n-1}\left[L\right]\right)$        $Stg_{n}\left[L\right]=LoadTo\left(Stg_{n+1}\left[E\right]\right)$    **end if**    $Stg_{n}\left[T\right]=Transform\left(Stg_{n}\left[E\right],App_{n}\right)$    sk=generatekey()                    ▹ Symmetric Key    $Metadata=[Stg_{n}\left[E\right],Stg_{n}\left[t\right],Stg_{n}\left[L\right]]$    mEnc=AesEncrypt(Metadata,sk)    de=genDigEnv(sk,OrgPubKey)            ▹ Digital envelope    LoadDE(dEnv,OrgId)    $DP_{V_{n}+1}=hash\left(Stg_{n}\left[T\right]\right)$    $registry(DP_{V_{n}+1},mEnc)$                 ▹ Registry method**end while**

To verify the accomplishment of agreements defined in smart contracts [36], we create a verifiability network which includes online and offline mechanisms for creating validation schemes for DPLs. This makes it possible to reconstruct or to describe the sequencing of the exchange of those products and their processing in pipelines or previously defined value chains.

The verificability network is based on Algorithms 1 and 2 that determine when online or offline mechanisms are invoked, respectively. The chosen mechanism receives a query for verifying the DPL of a given digital product version. That query can include requests for a partial or whole DPL. The mechanisms, both of them, return a structured file including the ETLs of a value chain used to manage the digital product indicated in a query plus a report of the accomplishment or violations of ETLs registered at smart contracts. This file is only delivered to end-users presenting valid credentials, which enable them to decrypt that file.
**Algorithm 2** Offline verificability process**Require:**$Stg_{i},OrgSecKey,idOrg$  $r=getEcryptedRegistry(Stg_{i})$                 ▹ Consult method  de=loadDE(idOrg)  sk=readDE(OrgSecKey)  d=decrypt(r,sk)  $DPL_{DP_{V_{i}}}=obtainHash\left(d\right)$  metadata=obtainETL(d)  etl=getETLHash(metadata)  $v=compare(DPL_{DP_{V_{i}}},etl)$  showMessage(v)

**Figure 3:**
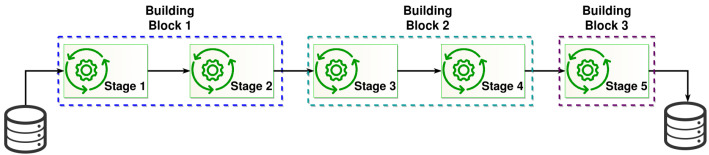
The abstraction of a value chain built by Building Blocks.

In the case of the online verification mechanism, a query to the blockchain by using the hash of a digital product is enough to recover the trace of that product ($DP_{V_{i}}$), which describes the ETL information from the original product to the last stage of the lifecycle of that product. The trace of a digital product ($DPL_{DP_{V_{o}}}$) delivers to valid users information describing either the whole or partial value chain of a given digital product (see a value chain of building blocks in Figure 3). This information is obtained by using an acyclic directed graph.

The ETL recovery information is basically a list of transactions of a given digital product, which is converted in a clear file when providing the valid credentials.

### 3.3. Offline Transaction Registration and Verification Using Fingerprinting

#### 3.3.1. Offline Verification by Using Fingerprinting

This offline verification is a key element in our traceability method for DPLM. Our method considers the usage of a fingerprinting technique that can be applied on different digital products. As a first approach, we have included in our method a collusion-resistant audio fingerprinting technique proposed in [11], which is based on a modified version of [37].

In this offline verification mechanism, the chain of each digital product ($DPL_{DP_{V_{i}}}$) is also embedded within the $DP_{V_{i}}$ by using the fingerprinting mechanism. This means that the end-users can retrieve the trace $DP_{V_{i}}$ by recovering the fingerprinting message from every digital product. In this case, the embedded message describes the accumulated transactions performed at the last stage that modified that digital product. The message recovery produces a set of concatenated hashes, which are sent to the online verification mechanism in the form of a query to obtain a report of accomplishment/violations of smart contract agreements/terms. This means that the hashes are converted into encrypted and descriptive metadata stored in a hash table (where the key is the identifier of each digital product and the value is the chain of ETLs captured during its DPL).

#### 3.3.2. Fingerprinting Design Principles

Pseudonoise (PN)-modulated orthogonal sequences are proposed in [37]. These orthogonal sequences can be obtained from a discrete cosine transform (DCT) or direct Fourier transform (DFT) basis. In the DCT case, each user is related to a DCT matrix column, which is defined in Equation (1).

(1) $$a\left(n,k\right)=c\left(k\right)\sqrt{\frac{2}{M}}\cos[(n+\frac{1}{2})\frac{k\pi }{M}]$$

where

(2) $$c\left(k\right)=\left\{\begin{array}{c} \begin{array}{ccc} 1/\sqrt{2} & \; & \mathrm{if}\;k=0\\ 1 & \; & \mathrm{otherwise} \end{array} \end{array}\right.$$

and *M* is the sample block length.

Therefore, the spread spectrum (SS) sequence for the *i*th user becomes:

(3) $$w_{i}=\beta \cdot \mathrm{pn}\left(s\right)⊗\mathrm{DCT}\left(i\right)\;$$

where β is a robustness factor, pn(s) is a PN sequence generated using an initial value s, s is a secret key, DCT(i) is the *i*th DCT matrix column and ⊗ is the element-wise multiplication. The sequence $w_{i}$ is embedded into the frequency components of the audio signals.

Unlike other watermarking applications, in the fingerprinting paradigm the detection is usually carried out in a non-blind fashion [38]; that is, the original signal is available to the detector. Under that condition, the sequence $\tilde{w_{i}}$ is obtained after subtracting the original sequence from the pirate copy. To carry out the detection, the sequence $\tilde{d}$ is obtained by applying the Inverse DCT to $\tilde{w_{i}}$, which is demodulated by the PN sequence pn(s), as follows:

(4) $$\tilde{d}=\mathrm{InverseDCT}\left(\mathrm{pn}\left(s\right)⊗\tilde{w_{i}}\right)$$

where InverseDCT(·) denotes a fast inverse discrete cosine transform algorithm. A threshold is necessary to determine the user under a statistical point of view. If $\tilde{d}$ is supposed to be $N(0,\sigma^{2})$ except for a fingerprinted component $\tilde{d_{k}}$, then it is possible to calculate a threshold *T* according to the probability of false detection $P_{fa}$ [37], as follows:

(5) $$P_{fa}\leq \frac{1}{2}\mathrm{erfc}(\frac{T}{\sqrt{2\sigma^{2}}})$$

where erfc(·) is the complementary error function, which is defined as:

(6) $$\mathrm{erfc}\left(x\right)=\frac{2}{\sqrt{\pi }}\int_{x}^{\infty }\exp\left(-u^{2}\right)\mathrm{du}$$

Therefore, the threshold is given by Equation (7),

(7) $$T=\sqrt{2\sigma^{2}}{\mathrm{erfc}}^{-1}\left(2P_{fa}\right)$$

where erfc${}^{-1}$(·) stands for the inverse complementary error function.

Grouping a set of users has been proposed in the literature as a solution to high computational costs [27,39]. The assumption behind this proposal is that users who have a similar background and region are more likely to collude with each other. The idea of introducing dependency between two SS sequences by exploiting the property of quasi-orthogonality of PN sequences is proposed in [37]. Therefore, the fingerprint is integrated by two spread spectrum sequences related to a group ID $w_{i_{g}}$ and a user ID $w_{i_{u}}$, as follows:

(8) $$w_{i_{g}}=\beta_{g}\cdot \mathrm{pn}\left(s\right)⊗\mathrm{DCT}\left(i_{g}\right)$$

where $\beta_{g}$ is the robustness factor for groups, pn(s) is a PN sequence generated with the secret key s, $\mathrm{DCT}(i_{g})$ is the *i*th basis vector that identified to the *i*th group and

(9) $$w_{i_{u}}=\beta_{u}\cdot \mathrm{pn}\left(i_{g}\right)⊗\mathrm{DCT}\left(i_{u}\right)$$

where $\beta_{u}$ is the robustness factor for users, $\mathrm{pn}(i_{g})$ is a PN sequence corresponding to the *i*th group and $\mathrm{DCT}(i_{u})$ is the *i*th basis vector that identified the *i*th user.

Then, the fingerprint assigned to the *j*th user of the *i*th group is confirmed by:

(10) $$w_{i,j}=w_{j_{u}}+w_{i_{g}}$$

The energy of the fingerprint is represented by

(11) $$\beta^{2}=\beta_{g}^{2}+\beta_{u}^{2}.$$

#### 3.3.3. Fingerprint Embedding

**Figure 4:**
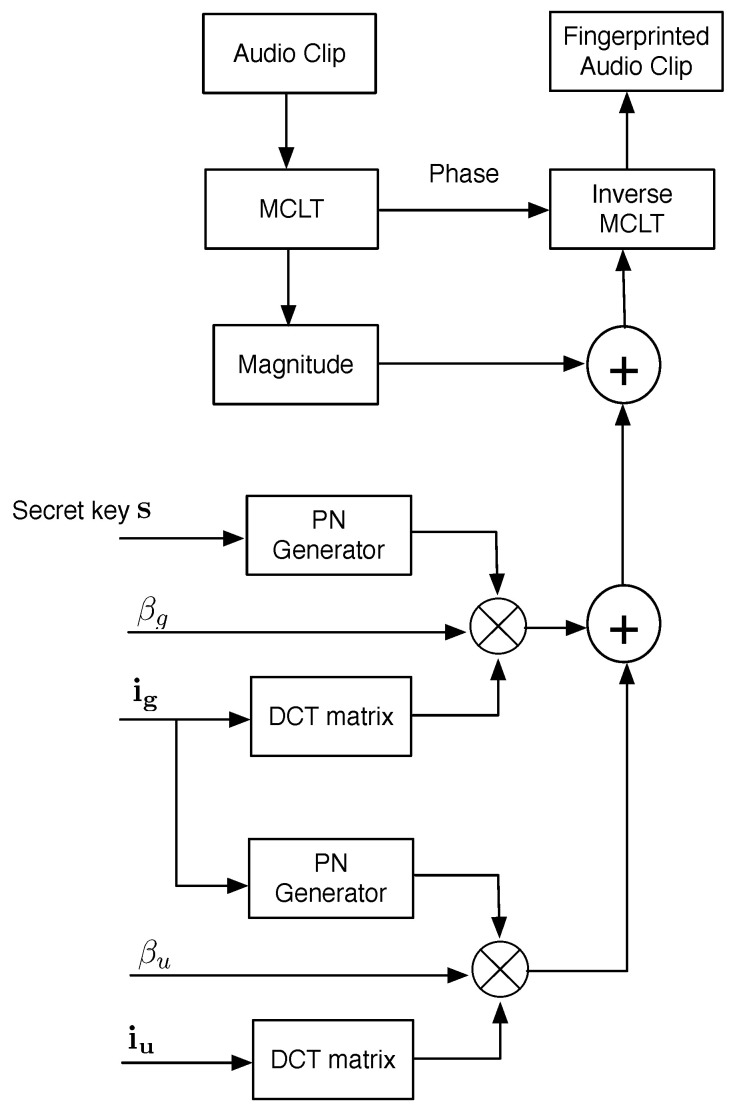
Fingerprint embedding system.

The fingerprint embedding system is illustrated in Figure 4 and described in the following.

First, a host audio signal is divided into frames of 2M samples per frame. Next, each frame is transformed using the modulated complex lapped transform (MCLT). Subsequently, both the magnitude and phase of MCLT are computed. The fingerprint is then added to the MCLT magnitudes while keeping phase without change. The additive technique is utilized for embedding as follows:

(12) $$\hat{X}=X+w_{i,j},$$

where $\hat{X}$ is the fingerprinted MCLT magnitude, X is the original MCLT magnitude and $w_{i,j}$ is the fingerprint assigned to the *j*th user of the *i*th group. Finally, inverse MCLT is applied to both processed magnitude and original phase to get the audio signal with a hidden fingerprint. The fingerprint conforms with Equation (10). The secret key *s* provides the system security in a symmetric-key fashion. The $i_{u}$ and $i_{g}$ variables represent the authorized user and its group, respectively. The PN-Generators produce pseudo-noise with a uniform distribution.

#### 3.3.4. Fingerprint Detection

From Equation (10), it is easy to see that a couple of detectors are required: one for the spread spectrum sequence related to group ID $w_{i_{g}}$ and the other for the user ID $w_{j_{u}}$. These detectors are derived from Equation (4) as follows.

**Figure 5:**
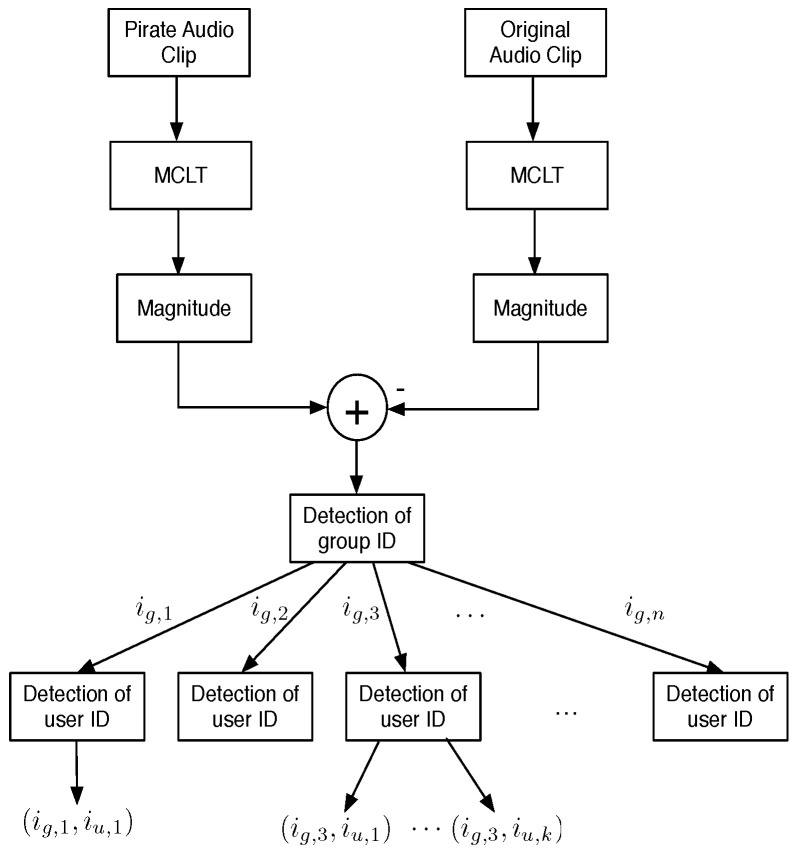
Colluder detection system.

For group ID detection:

(13) $${\tilde{d}}_{g}=\mathrm{InverseDCT}\left(\mathrm{pn}\left(s\right)⊗{\tilde{w}}_{i,j}\right)$$

and for user ID detection:

(14) $${\tilde{d}}_{u}=\mathrm{InverseDCT}\left(\mathrm{pn}\left(i_{g}\right)⊗{\tilde{w}}_{i,j}\right)$$

with thresholds, $T_{g}$ and $T_{u}$, derived according to Equation (7), as follows:

(15) $$T_{g}=\sqrt{2\sigma_{g}^{2}}{\mathrm{erfc}}^{-1}\left(2P_{fa_{g}}\right)$$

(16) $$T_{u}=\sqrt{2\sigma_{u}^{2}}{\mathrm{erfc}}^{-1}\left(2P_{fa_{u}}\right)$$

where $P_{fa_{g}}$ and $P_{fa_{u}}$ are given false positive probabilities for the group and user ID detection procedures, respectively. $\sigma_{g}^{2}$ and $\sigma_{u}^{2}$ are the variance of the group and user ID detection sequences, respectively. Figure 5 shows the fingerprint detection system.

#### 3.3.5. Group ID Detection

**Figure 6:**
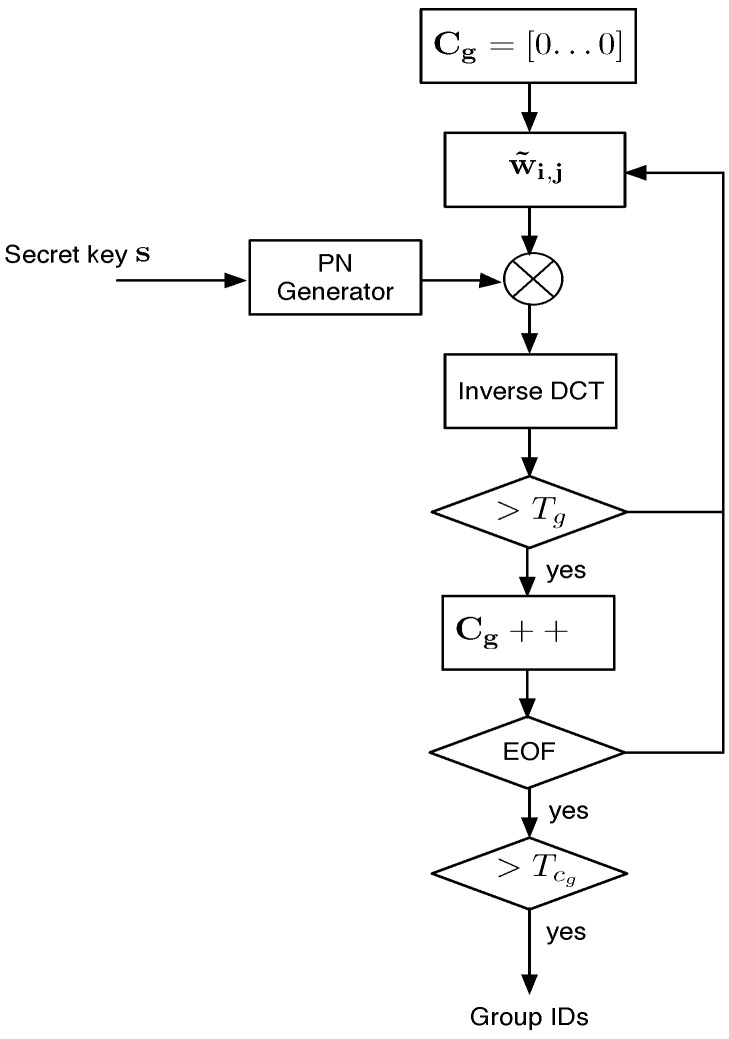
Group ID detection system.

Figure 6 shows the group ID detection system. For each available MCLT coefficient block, group detection is carried out according to the threshold, $T_{g}$, as described in Equation (15). For the whole pirate audio clip, there is a counter vector $C_{g}$ that registers the number of times that each component of ${\tilde{d}}_{g}$ exceeds $T_{g}$. Instead of the group ID detection in each block where the threshold is computed assuming a Gaussian distribution, the threshold, $T_{c_{g}}$, for detection in the counter vector $C_{g}$ must consider other distributions because the lower limit of that distribution will always be zero. In [11], it is proposed that $C_{g}$ holds a half-Gaussian distribution. Therefore, the threshold, $T_{C_{g}}$ for a $P_{fa}$ given for a group ID detection in a pirate audio clip can be computed as follows:

(17) $$T_{C_{g}}=\sqrt{2\sigma_{C_{g}}^{2}}{\mathrm{erfc}}^{-1}\left(P_{fa}\right)$$

where $\sigma_{C_{g}}^{2}$ is the variance of $C_{g}$.

#### 3.3.6. User ID Detection

**Figure 7:**
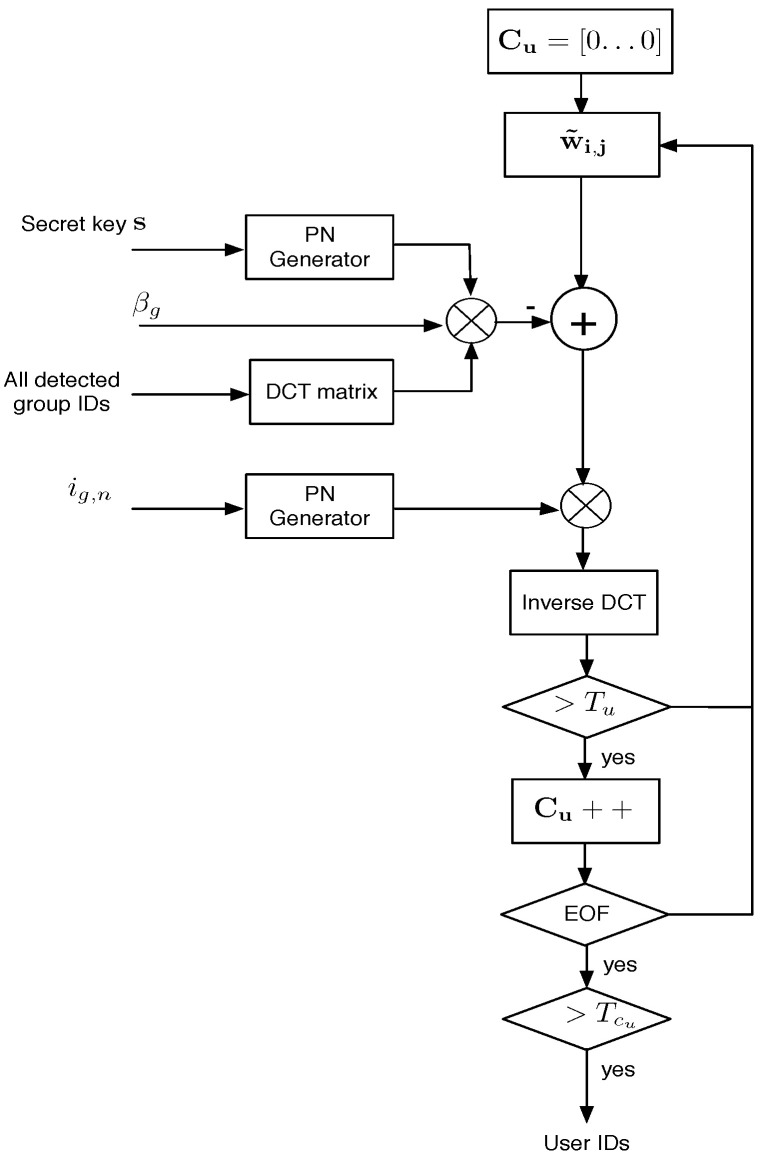
User ID detection system.

Figure 7 shows the user ID detection system. In similar form to group ID detection: for each available MCLT coefficient block, user detection is carried out according to the threshold, $T_{u}$, as described in Equation (16). For the whole pirate audio clip, there is a counter vector $C_{u}$ that registers the number of times that each component of ${\tilde{d}}_{u}$ exceeds $T_{u}$. Counter vector $C_{u}$ is modeled as a half-normal distribution and the corresponding threshold $T_{C_{u}}$ for a given $P_{fa}$ is calculated according to:

(18) $$T_{C_{u}}=\sqrt{2\sigma_{C_{u}}^{2}}{\mathrm{erfc}}^{-1}\left(P_{fa}\right)$$

where $\sigma_{C_{u}}^{2}$ is the variance of $C_{u}$. To improve user ID detection, the interference due to group ID is previously removed.

### 3.4. Security Management in the Verifiability of Transactions

The following are the security services included in this traceability method to manage/mitigate risks when verifying transactions and verifying accomplishment of agreements in DPLM:

- Privacy, confidentiality and access controls: These services are implemented in the DPLM middleware by using different cryptosystems to ensure the information is registered in the blockchain. AES cryptosystem is used to encrypt the information about the paths where data have been extracted by a stage and the path wherein outcomes were deposited as well as the timestamps that provide information about the productivity among the involved stages in the DPLM. The key management and delivery is performed by using digital envelopes, which operate at the creation of the stages.
- Integrity: The Sha256 algorithm is used as a method in the smart contracts to create the hashes of the input and outcomes as well as to produce the hash of all the information stored in the block chains (input and outcome hashes, timestamps and information required by smart contracts). This means the integrity of the inputs and outcomes are provided by digital envelopes and this information is registered both in the blockchain and within the contents by using the fingerprinting method. The verifiability process is thus supported by integrity service at the registration phase and by the blockchain querying at the online phase as well as by the fingerprinting at the offline phase.

## 4. Experimental Evaluation

This section describes the experimentation carried out to test our proposed traceability method in a supply chain of digital contents. For this case of study, an audio DPLM cloud service was implemented to represent the supply chain of digital contents (audio files). The purpose of the experimentation is to analyze how the different modules and elements involved in the audio DPLM service (noise reduction, fingerprinting, verification network or blockchain, content distribution network) impact the performance of the overall service. The prototype considers an environment of multiple value chains for digital contents deployed over the same administrative domain, wherein a verifiability network (or blockchain network) is in charge of recording the transactions executed in every process (i.e., every stage) involved in the value chains as well as tracing the transfer of digital assets from one source to one destination in the content distribution network.

### 4.1. Experiment Methodology

This experimentation is based on the use of four stages of digital audio processing, each of them adding value to obtain the final content. It should be noted that the insertion of a digital fingerprint into the audio is the primary process within these. Each stage is described below.

1. Raw material. In this stage the raw material provided by the producer is received and stored and the processing begins within the corresponding value chain, generating the identifiers, orders and elements necessary for the control, management and shipment of the assets through each of the flows.
2. Noise reduction. Noise cleaning processing on each of the provided audios. This aims to minimize these unwanted sounds while preserving the original signal as much as possible.
3. Fingerprint insertion. Process of inserting direct information to the digital audio content which helps to obtain better levels of security and privacy.
4. Compression. Process of reducing the size of the information to occupy less storage space, without affecting the content.

**Figure 8:**
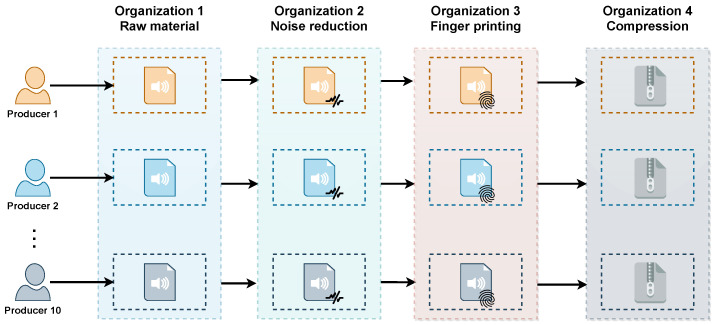
Controlled experimentation with audio fingerprinting process in 10 value chains.

Two experiments were generated, varying the number of chains. In the first experiment, one value chain was used and in experiment two, ten value chains were constructed, assigning each organization a different processing. In addition, as shown in Figure 8, different producers were defined to initialize each of the treated chains. It can be seen that for each of the producers a specific value chain is generated in which all the files provided by that entity will be processed in the same way until a value-added product is obtained for the consumer. It can also be noted that for each of the processes involved there is an organization that provides these services.

**Figure 9:**
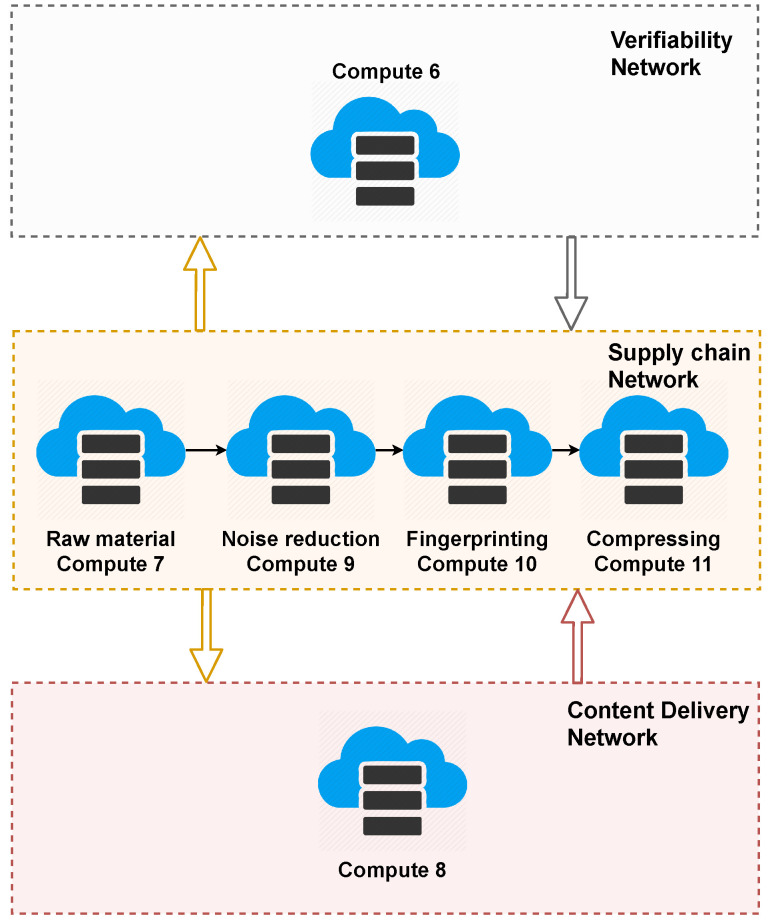
Distribution of services of the three networks of the solution.

For the purposes of the development of this evaluation scenario, the services of each network were distributed as shown in Figure 9, using six servers from the Cinvestav–Tamaulipas private cloud.

Several experiments were carried out in this scenario with variations in the number of files to be processed as well as in the number of value chains. It is important to mention that the content distribution was performed in batches.

### 4.2. Implementation Details

We propose the deployment and evaluation of an audio DPLM cloud service to represent the supply chain of digital audio files to analyze the effect of the use of different modules and elements involved in the audio DPLM service. The audio dataset consisted of CD-quality audio clips; each clip had a sampling rate of 44,100 samples per second and had one channel. The digital audio processing stages (raw material, noise reduction, fingerprint insertion and compression) were implemented in C programming language. The creation of a supply chain and the communication with the framework using Hyperledger Fabric were implemented with C programming language and Python. An AES-CBC cipher with a 256 bit key was implemented in C programming language. A digital envelope based on elliptic curve cryptography (ECC) was developed in Java and C languages. All the process, stages and APIs were encapsulated into Docker container images in order to deploy Docker virtual containers in multiple infrastructures like the cloud.

### 4.3. Single Value Chain Experiment

The experiment was developed by generating a single value chain considering all the stages mentioned in the scenario evaluation methodology. Three different configurations were considered varying the number of assets to be processed: one, ten and one hundred. The purpose of this experiment is to observe the impact of workloads considering that all services are distributed.

**Figure 10:**
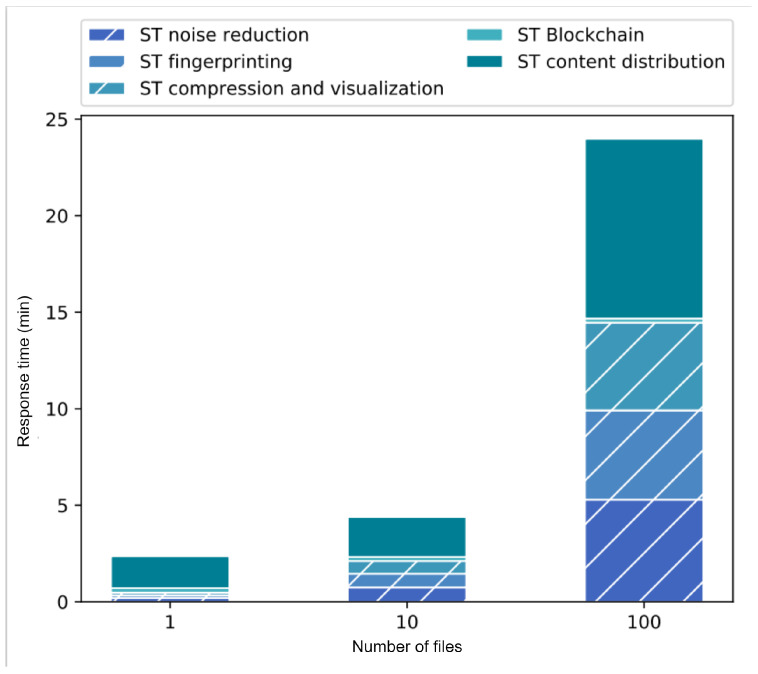
Response time of a value chain with three different configurations.

Figure 10 shows the response time of the chain considering the variation in the number of assets to be processed. These response times are composed of the service time of each stage (ST): noise reduction, fingerprint insertion, compression and visualization, as well as the service times of the verifiability network (or blockchain) and the content distribution network (i.e., sending and receiving of assets). The vertical axis shows the response time of the whole chain and the horizontal axis shows the different file quantity configurations. It can be noted that the component that has the greatest impact on the solution is the distribution of contents and it can also be seen that the service time of the verifiability network is constant regardless of the number of files. That is because the information and the amount of queries required by the verifiability network is almost the same for each of the assets in each of the stages, which means that the time spent in this query action is semi constant.

If we analyze the behavior of the service times of each of the stages, we can conclude that they are a function of both the building block management and the applications they contain. Since the applications used in this scenario are lightweight, the service times of each of the stages are similar in each of their configurations. It is noted that the most time-consuming application is the one that performs the noise reduction process since it uses a larger number of operations on the asset. In addition, the solution uses pipeline processing, which impacts the response time of each of the components because they process each of the files individually and the requests are queued within each of the stages.

In conclusion, the overhead produced by the traceability process throughout the digital content value chain is low compared with the workload produced by the different elements (stages) that compose the chain. This overhead decreases as the number of digital assets to be processed increases.

**Figure 11:**
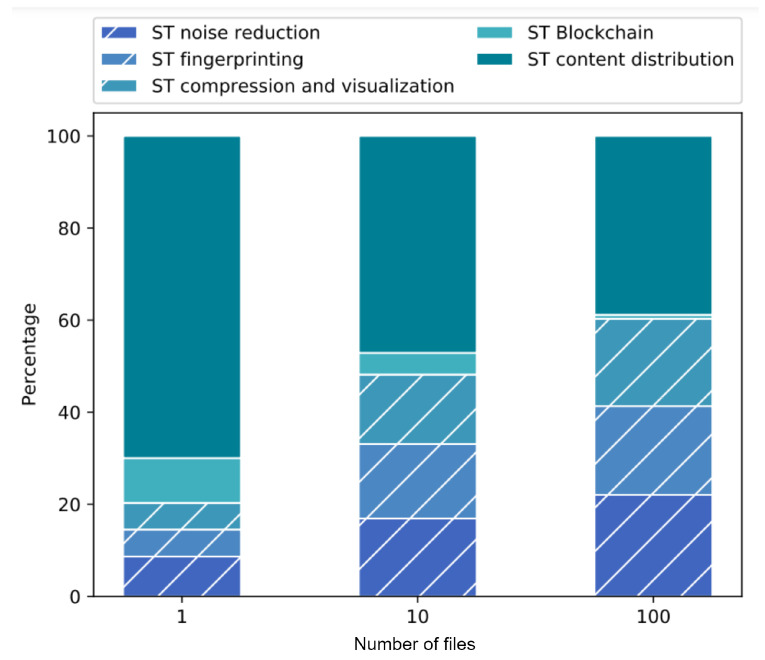
Percentage of time used by each component in a value chain.

Figure 11 shows the impact of every processing stage in the value chain evaluated by percentages. With this representation, it can be seen again that the greatest impact on the chain is the time to send and receive files, it can also be seen that this decreases with a lower number of digital assets (files). In other words, the higher the number of files, the longer the service time required for each of the stages included in the chain. The percentage of use of the verifiability network decreases as the number of files increases because it has a constant service time per asset. In addition, by taking the relation of the elements of the chain, the difference in time required for each of the stages can be noted. It is concluded that this variation is mainly due to the actions that each application must perform to add value to the product, since the management (verifiability) activities per stage are the same and the time required for this is constant. In this experiment, the service time in the processing stages involved in the value chain are similar. However, the content distribution process is the one that takes longer.

### 4.4. Ten Value Chain Experiment

A variation in the number of value chains in the solution was carried out to observe the behavior that this generates in the proposal.

This experiment was developed by generating ten independent value chains considering all the stages mentioned. In order to obtain a comparison with the previous experiment, the same variation in the number of files to be processed was considered; in this sense, the experiments were carried out with one, ten and one hundred files. The purpose of this experiment is to observe the impact generated by increasing the number of value chains that each of the organizations will serve in order to evaluate whether this significantly affects the execution times of each of the components involved.

**Figure 12:**
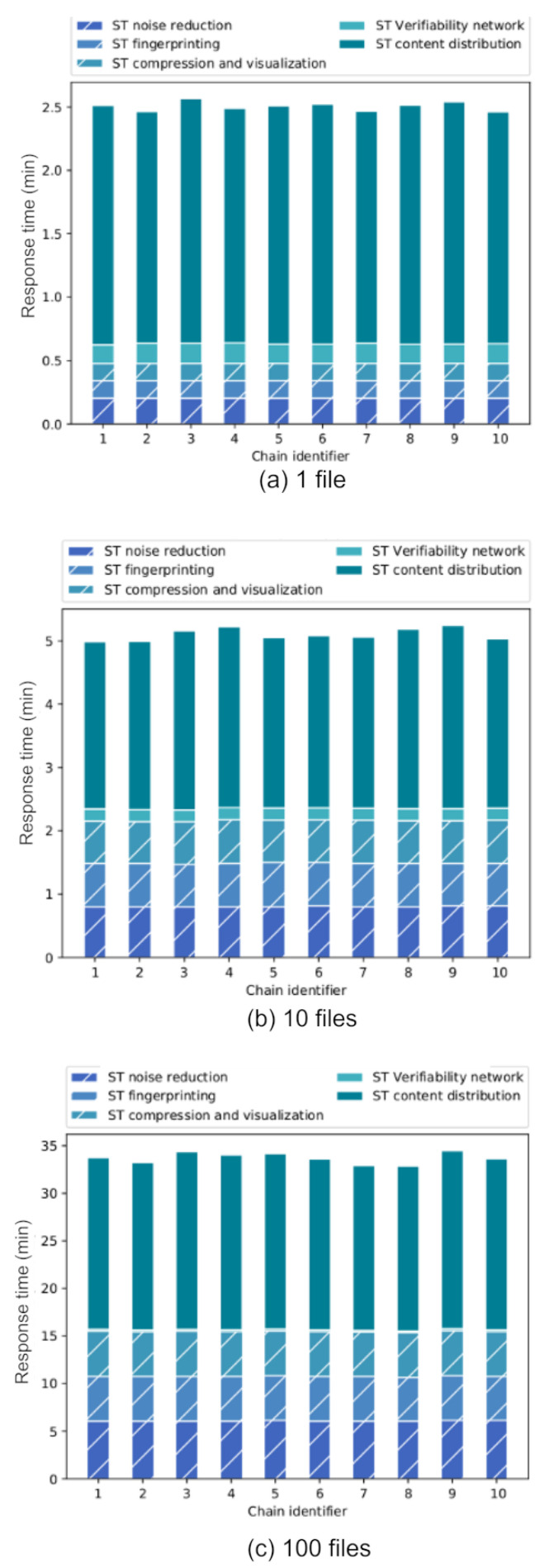
Absolute response time per value chain divided by components using (**a**) 1 file, (**b**) 10 files and (**c**) 100 files.

Figure 12 shows three graphs corresponding to the results obtained for each evaluated configuration. Each of them shows on the vertical axis the response time per chain in minutes and on the horizontal axis the different value chains launched. Figure 12a shows the results obtained when evaluating the processing of a single file; in Figure 12b, ten files were evaluated; Figure 12c shows the evaluation of one hundred files per chain.

It is important to highlight the fact that each of the chains works independently with self-management of the products that each of the organizations receives and therefore there is an implicit parallelism that helps to ensure that the response times of the chains evaluated do not differ significantly.

Once again, it can be seen that the component with the greatest impact on the solution for all configurations is the time for sending and receiving the asset (content distribution). In addition, the service times of the blockchain network are close for each of the configurations, because the queries and information sent for each of the chains have the same structure. It is worth mentioning that the traceability feature was set for each of the processed assets.

The behavior observed for each of the chains for the configuration of one and ten files is similar to the behavior observed in the experimentation with a single chain, varying only by 6.1% and 10.7%, respectively, which shows that in these cases the use of a greater number of value chains does not significantly affect the performance and response time of the solution. Considering this behavior and as far as the computational resources allow it, our solution could provide the service with the same quality to a greater number of flows.

On the other hand, for the configuration of 100 files per chain, a difference of approximately 28.9% in service time degradation is observed compared to the use of only one chain. This indicates that the concurrency of chains in the solution affects the service time when increasing the number of assets to be processed. This point will be compared with the past experimentation in terms of performance to analyze the feasibility of increasing the number of chains with a greater number of files.

**Figure 13:**
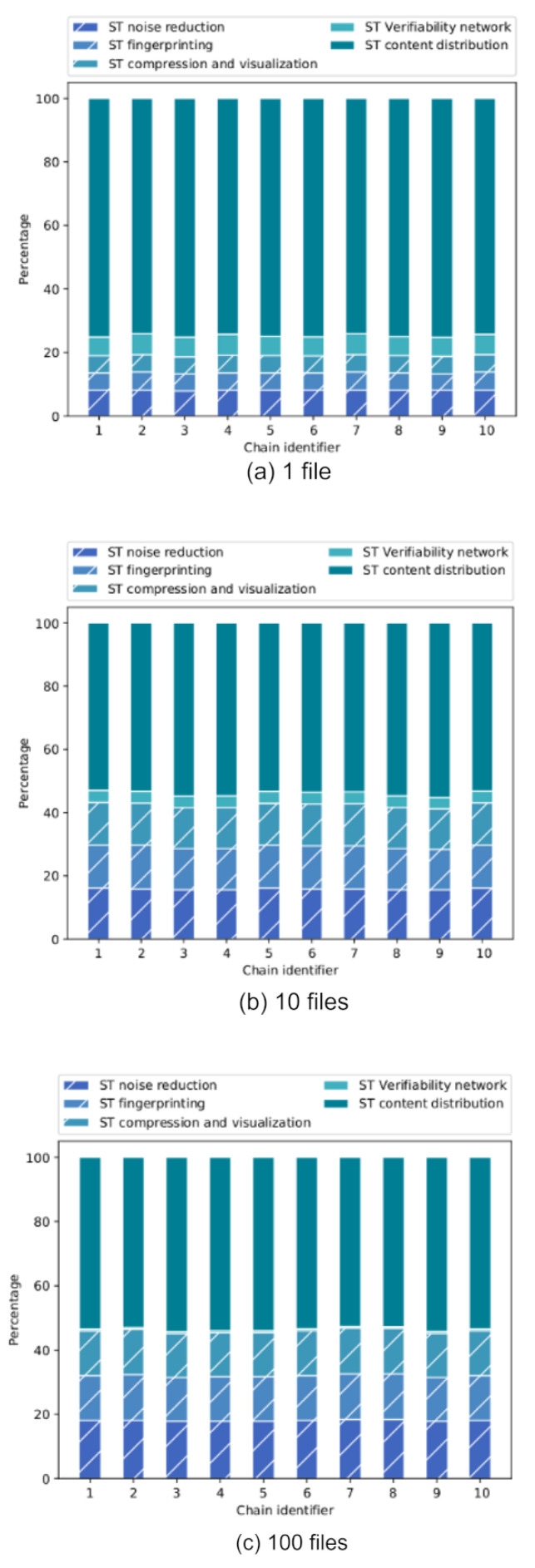
Percentage of time of each component per value chain using (**a**) 1 file, (**b**) 10 files and (**c**) 100 files in an experiment with ten chains.

Figure 13 shows the proportion of time that each chain component requires. It can be seen that there is no significant improvement in the time of the distribution component for the one-file and ten-file configurations, since the times required for each of the stages are stabilized (they are very similar) and are proportional to the absolute response time of each chain. On the other hand, it is not convenient to process only one file in the batch because in this case the distribution time takes more than 70% of the total time required in the chain.

**Figure 14:**
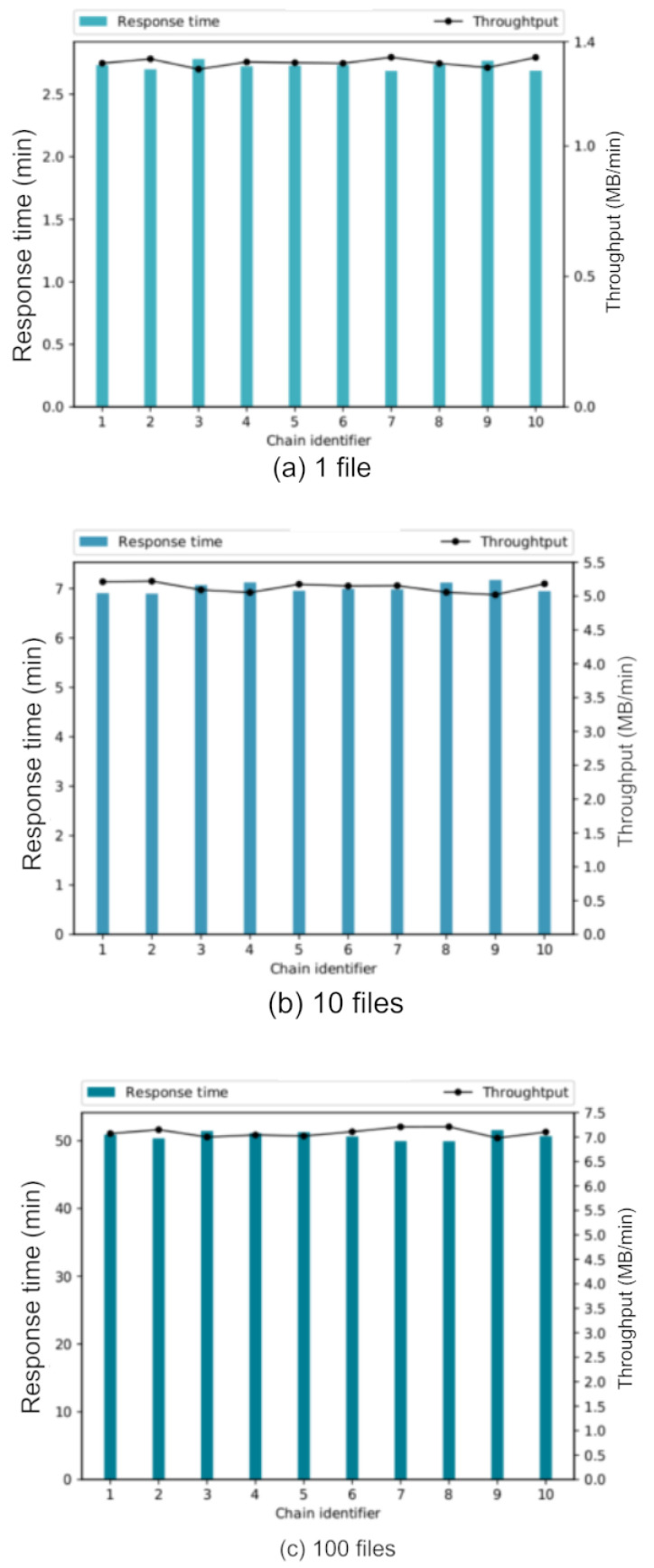
Throughput per configuration in a ten-value chain experiment using (**a**) 1 file, (**b**) 10 files and (**c**) 100 files.

Figure 14 shows the throughput per chain of each of the configurations used. The response time in minutes is shown on the left vertical axis, on the right vertical axis is the throughput (MB/min) and on the horizontal axis is shown the identifier of each of the chains involved.

Figure 14a shows information about the experiment with a single file, Figure 14b shows the results of the experiment with ten files and Figure 14c with one hundred files. It can be noted that the performance increases as the number of files to be processed increases.

Figure 14 allows us to conclude that the increase of files per chain is feasible because the general throughput increases significantly, from 1.4 MB/min (for the case of one file) to 7 MB/min (for the case of 100 files).

Logically, the processing time increases as the number of files increases, but there is a relationship between the time and the amount of data to be processed; therefore, with a configuration of a larger number of files, the performance is increased and it can be affirmed that our solution is feasible in parallel processing of digital assets with supply chains.

It should be noted that this behavior is observed using ten independent chains with the IT infrastructure mentioned. More concurrent processing chains were launched, showing similar results, which are not included in this paper. However, it is possible that there is a restriction in IT capacities in terms of impacting the performance of the overall system.

### 4.5. Experiments Summary

**Figure 15:**
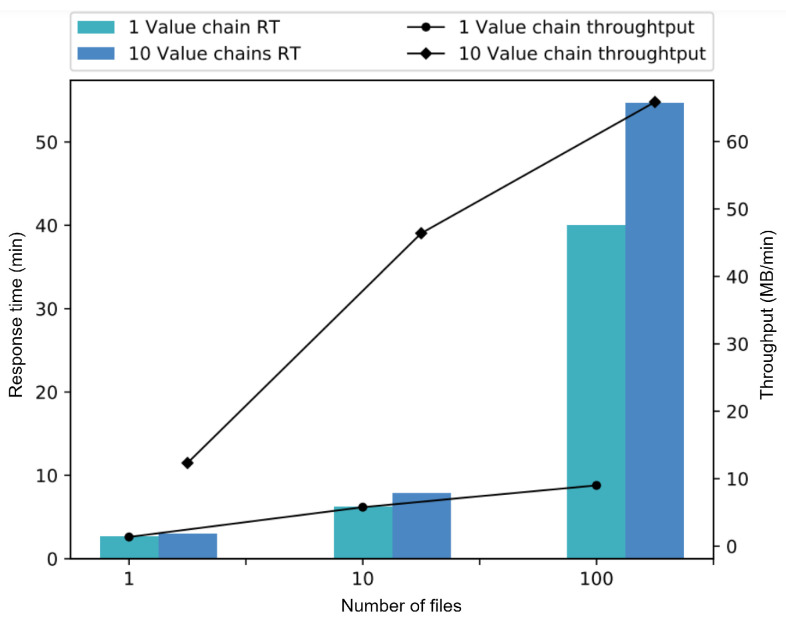
Comparison between experiments with one and ten value chains.

To provide a general vision of the behavior of the SC service, the main results obtained in the experiments are gathered and analyzed in one graph. Figure 15 shows the response time and throughput metrics by supply chain. It already includes all the value chains processed by each experiment.

The left vertical axis shows the response time per supply chain and the right vertical axis shows its throughput. The horizontal axis shows the configuration in relation with the number of files processed in each experiment. The color indicates the number of value chains (1 and 10). By analyzing Figure 15, it is worth noting that for all variations in file quantity there is a significant performance improvement in the solution for the ten chain use case, this is because our SC service executes each chain independently and allows parallel processing. It is therefore recommended to use a larger number of value chains to increase the performance of the solution as long as the computational resources allow it and the user’s needs are adapted to it.

## 5. Conclusions

In this paper, we have presented the design, development and validation of a novel traceability method for DPLM. On the one hand, the method uses a blockchain-based verification process to register the transactions performed by the organizations involved in the processing of digital products. On the other hand, our approach integrates a fingerprinting scheme to verify the integrity of digital product content. As a proof of concept, our DPLM approach was deployed in a private cloud computing infrastructure for experimental purposes. A case study based on the DPLM of digital audios was carried out, deploying an end-to-end solution implementing BCT with the detection of users tampering with digital products, on a set of service supply chains, composed of a set of stages, using different configurations.

The following findings were revealed by the results of the experiments. The overhead produced by the traceability process (verifiability network) throughout the digital content value chain is low compared with the workload produced by the different elements (stages) that compose the chain. The percentage of use of the verifiability network decreases as the number of digital assets increases because it has a constant service time per asset. The service time variation among each of the stages included in the chain is mainly due to the actions that each application (used in each stage) must perform to add value to the digital asset, since the management activities (verifiability) per stage are the same and the time required for this is almost constant. The deployed chains, using our traceability approach, work independently with self-management of the products that each of the organizations receives and therefore there is an implicit parallelism that helps to ensure that the response times of the chains evaluated do not differ significantly. In this sense, the use of a greater number of value chains does not significantly affect the performance and response time of the solution. Considering this behavior and as far as the computational resources allow, our proposal could provide the SC service with the same quality, using a greater number of flows than those used in the experiments. The concurrency of chains in the solution affects the service time when increasing the number of assets to be processed. Experiments showed that it is not convenient to process only one digital asset in batch because the distribution of digital content becomes the major time consuming element. With a single file in the value chain, this element could consume more than 70% of the total service time. However, experiments showed that the increase of files per chain is feasible because the general throughput increases significantly, e.g., from 1.4 MB/min (for the case of one file) to 7 MB/min (for the case of 100 files). Logically, the processing time increases as the number of files increases, making evident a relationship between the time and the amount of data to be processed. With a configuration of a larger number of files, the overall performance increases, making our solution feasible in parallel processing of digital assets with supply chains. It is therefore recommended to use a larger number of value chains as long as the computational resources allow it and it responds to the user’s needs.

## Data Availability

Not applicable.
